# Evaluation of the Biological Response to Coating 3D-Printed PLA Scaffolds with Coaxial Gelatin-Based Electrospun Fibers

**DOI:** 10.3390/biomimetics11050356

**Published:** 2026-05-20

**Authors:** Cristian Enrique Torres-Salcido, Aída Gutiérrez-Alejandre, Jesús Ángel Arenas-Alatorre, Janeth Serrano-Bello, Vincenzo Guarino, Marco Antonio Alvarez-Perez

**Affiliations:** 1 Laboratorio de Bioingeniería de Tejidos, División de Estudios de Posgrado e Investigación (DEPeI), Facultad de Odontología, Universidad Nacional Autónoma de México (UNAM), Ciudad Universitaria, Coyoacán, Mexico City 04510, Mexico; cristiantorressalcido@comunidad.unam.mx (C.E.T.-S.); janserbello@fo.odonto.unam.mx (J.S.-B.); 2 Unidad de Investigación en Catálisis (UNICAT), Departamento de Ingeniería Química, Facultad de Química, Universidad Nacional Autónoma de México (UNAM), Mexico City 04510, Mexico; aidag@unam.mx; 3 Laboratorio 113 Síntesis de Nanomateriales Magnéticos, Departamento de Materia Condensada, Instituto de Física, Universidad Nacional Autónoma de México (UNAM), Ciudad Universitaria, Coyoacán, Mexico City 04510, Mexico; jarenas@fisica.unam.mx; 4 Institute of Polymers, Composites and Biomaterials, National Research Council of Italy, Mostra d’Oltremare, Pad.20, V.le J.F. Kennedy 54, 80125 Naples, Italy

**Keywords:** core/shell nanofibers, coaxial electrospinning, 3D-printing, biocompatibility, bone regeneration

## Abstract

Bone grafting remains limited, and the strategies to design even more structurally complex scaffolds—able to reproduce the hierarchical architecture of bone extracellular matrix—are rapidly growing. In this study, we report the fabrication of a hierarchically structured scaffold produced by layering poly(ε-caprolactone)/gelatin (PCL/Gt) or poly(lactic acid)/gelatin (PLA/Gt) electrospun nanofibers via coaxial electrospinning onto 3D-printed poly(lactic acid) (PLA) scaffolds via fused deposition modeling (FDM). After the printing process, PLA disks (10 × 1 mm, 20% infill, ~80% porosity, pore size ~1.57 mm) were coated with core/shell (PCL/Gt, PLA/Gt) fibers to investigate the in vitro interfacial response of osteoblasts in comparison with monocomponent fibrous coatings (PCL, PLA, Gt). SEM and TEM confirmed that core/shell fibers exhibited bead-free morphologies, with a significant reduction in fiber diameter (≈287–316 nm) and higher interfibrillar porosity compared to monocomponent fibers. FTIR and thermogravimetric analyses indicated the presence of hydrogen bonding between the polyester and gelatin, and the absence of residual solvent after deposition. At the same time, water contact angle measurements confirmed an increase in hydrophilic properties from 80–86° to 120° ascribable to the presence of gelatin. Accordingly, in vitro response of human fetal osteoblasts (hFOB 1.19) exhibited an evident improvement in the case of Gt-based fibrous coatings (i.e., PCL/Gt and PLA/Gt) in terms of early adhesion (4–24 h) and metabolic activity from 3 to 21 days, cell spreading into star-shaped morphologies, formation of extracellular matrix, and mineral phase deposition. In more detail, a remarkable increase in alkaline phosphatase activity was observed in Gt-based coaxial coatings from day 7 onward, with the highest values recorded for PLA/Gt. Overall, we demonstrated that the Gt-based coaxial fibrous coating provided a mix of topological and biochemical cues that synergistically promoted key osteoblast activities at the interface, supporting the regeneration of new bone tissue in highly tailored 3D-printed scaffolds, thus suggesting a promising strategy for personalized regenerative medicine.

## 1. Introduction

Bone tissue has important biological functions, including protecting vital organs (such as the brain, lungs, and heart) from trauma. Additionally, bone marrow produces blood cells, and bones play a role in mineral homeostasis and endocrine regulation [1]. Bones also provide support for the body and facilitate movement [2]. However, these functions may be compromised due to several factors, including traumatic injuries, medical surgery, congenital and degenerative diseases [3,4]. Auto-grafting remains the “gold standard” for reconstructing critical-size skeletal defects. Nevertheless, limitations such as low bioavailability and complications associated with donor sites and surgical complications are significant challenges [5].

Current alternatives for bone repair include the development of biomaterial-based scaffolds through bioengineering technologies, such as polymer-based scaffolds, to avoid the complications of conventional bone grafting procedures, which lack an effective strategy for repairing large bone defects [4,6]. For this purpose, aliphatic polyesters, such as poly(lactic acid) (PLA) [7], poly(ε-caprolactone) (PCL) [8], poly(lactic-co-glycolic acid) (PLGA) [9], can be preferentially used due to their ease of processability, chemical stability, and mechanical performance. While non-degradable polymers, such as polyaryletherketones and polyvinylidenfluorides, are typically used as dental [10] and orthopedic surgery implants [11], aliphatic polyesters are typically addressed to scaffold manufacturing due to their highly modulable biodegradation properties and bioabsorbability, which help avoid repeated surgery procedures, thus reducing healthcare costs and implant-associated complications [12]. This allows, to a large extent, the mimicking of the structural properties of bone tissue by providing mechanical support and a suitable environment for optimal biological activity, thereby promoting cell attachment, proliferation, and differentiation without causing cytotoxicity or an inflammatory response [5,13]. The main challenge in scaffold development for bone tissue engineering (BTE) applications is to generate 3D structures that serve as artificial platforms with high mechanical properties, have non-cytotoxic properties, allow cell adhesion and proliferation, and finally, they must promote or lead differentiation into osteoblasts, enabling the formation and maturation of the mineralized bone matrix [14].

In order to reproduce the complex architecture of bone, several techniques have been employed for scaffold fabrication, including air jet spinning [15,16], solution blow spinning [17], gas foaming [18], freeze drying [19], electrospinning [20,21,22], electrospraying [23], salt leaching [24], and three-dimensional printing [25,26].

In the last decade, 3D printing technology has become a powerful tool in the biomedical field for fabricating scaffolds for bone tissue engineering (BTE) due to the accuracy of the additive manufacturing process in producing replicable architectures [27].

This technique allows us to create a three-dimensional scaffold using a pre-established computer-aided design (CAD), enabling the manufacture of highly complex 3D structures that mimic bone architecture and macrostructural organization [3,16]. Among 3D printing methods, material extrusion-based techniques, such as fused deposition modeling (FDM), have been widely used the fabricate scaffolds for bone regeneration. This approach is based on a layer-by-layer additive deposition process that allows assembling thermoplastic polymer filaments into the final product based on a 3D-designed model [28,29,30,31,32,33].

FDM technologies are a solvent-free and low-cost manufacturing process, and are also faster than other scaffold processing techniques [30]. However, they often have limited resolution, resulting in relatively large pore sizes (>100 µm); they also produce flat surfaces via polymer thermoforming, which reduce the number of cell–material adhesion sites [34]. To address these issues, some investigations focus on the modification of 3D printing technologies [35], such as melt electrowriting [36], or the combination of them with other scaffold manufacturing techniques, including air jet spinning (AJS) [16] and electrospinning (ES) [37]—providing new alternatives to achieve complex micro/nanostructures and more accurately mimic the extracellular matrix (ECM) of native tissues.

The ES technique enables the formation of micro- and sub-micrometric fibrous structures by applying a high voltage to a polymer solution [38,39]. The electrostatic repulsion forces at the needle tip lead to the formation of a “Taylor cone” and drive the deposition of fibers onto the grounded collector [40]. This allows the formation of fibrous structures with a wide range of fiber diameters (5–50 µm), a high specific surface area (1–100 m^2^/g), and interconnected porosity (80–90%) [41]. These features facilitate the transport of nutrients, cellular waste, and gases, such as oxygen and carbon dioxide [42]. The nanofibers produced by the ES technique mimic the nanoscale fibrillar structure of the ECM [43,44], making them suitable as coating surfaces for 3D scaffolds and modulating the micro- and nanotopography [45].

There are many ES process configurations based on the use of traditional single-nozzle, multi-needle, and coaxial methods [40]. In the last decade, the coaxial method has been particularly useful for drug incorporation, enabling controlled release through the customization of core/shell architectures [46]. Conversely, a single-nozzle configuration has been preferentially used for the fabrication of cell-loaded scaffolds. However, the lack of 3D structural organization at the macroscale level poses significant problems for its application in BTE [34].

Recent studies proposed different approaches based on the synergistic use of ES and 3D printing technologies to overcome some limitations of 3D printing alone in terms of structural resolution and accuracy. By their combination, 3D printing allows obtaining a scaffold with reproducible geometry on a macro-level, while ES allows reproducing an ECM-like structure at the cell interface [47,48]. Accordingly, recent studies have proposed the fabrication of PCL scaffolds with a dual-dimensional scale structure, processed by FDM and ES, demonstrating the ability of mesh topography and electrospun nanofiber alignment to promote cell seeding efficiency, cell spreading and fast colonization of the scaffold [49]. Similar studies combining Gelatin nanofibers and PLLA 3D-printed scaffolds highlighted the ability of gelatin electrospun fibers to improve cell adhesion and proliferation on the surface of the 3D-printed scaffold with pore gradients used for implant fixation in the case of subchondral bone reconstruction [50].

In this context, an accurate selection of biomaterials is crucial to satisfy the main conditions of processability required to obtain tailored scaffolds for BTE applications in terms of structural and biological properties [51]. Polyesters such as PCL and PLA were usually preferred to fabricate scaffolds via FDM and ES due to their low cost, ease of processing, and wide availability [33,52,53]. It is well known that PLA and PCL are biocompatible [54], mechanically stable in vitro [55], and both approved by the U.S. Food and Drug Administration (FDA) for use in the fabrication of medical devices. The higher stiffness and strength modulus of PLA [55], due to its peculiar macromolecular structure, combined with a faster degradation rate than PCL, its use tends to be favored for bone grafts, while PCL is preferred for osteochondral defects [56]. Despite their suitability for load-bearing applications, they inherently lack cellular recognition sites; therefore, they must be combined with natural biopolymers to enhance biological recognition [57,58,59].

To date, they are currently combined with bioactive proteins such as collagen [60,61,62,63,64], fibrinogen [65], and gelatin (Gt) [59,66,67,68,69]. Among them, Gt, obtained by hydrolyzing collagen, was selected for the presence of adhesion sites of RGD (Arg-Gly-Asp) amino acid sequences, able to promote integrin-mediated cell attachment to substrates. Due to the solubility in aqueous solutions, it is often treated by cross-linking agents, such as EDC/NHS or genipin [41], to stabilize mechanical properties and minimize the solubilization under physiological conditions. Indeed, uncross-linked Gt tends to promote early focal adhesions, by the trigger of carbodiimide reaction of carboxylate side chains in amino acid residues (i.e., Asp), leading to RGD sites unavailable to establish integrin-mediated cell binding [70].

Our group recently optimized the fabrication of a tubular scaffold made of PLA via 3D printing and coated it with a thin layer of PLA nanofibers via the air-jet spinning (AJS) technique, demonstrating that nanofiber coatings improved cell attachment, proliferation, and osteoblast cell morphology compared with uncoated tubular systems [16].

Starting from the current experimental evidence, this work proposes to investigate the biological response for 3D-printed PLA scaffolds coated with Gt-based coaxial electrospun nanofibers (CNFs). The combination of 3D printing and ES techniques contributes to expanding the knowledge of the development on advanced platforms with complex structures and multiple size scales, allowing the leveraging of the benefits of the nanofibrous coatings to enhance the surface properties of 3D-printed PLA scaffolds for early-stage guidance in bone tissue restoration.

The resulting multicomponent scaffold showed a superior interfacial property, able to support in vitro bone tissue growth. The main findings show that Gt-based CNFs onto 3D-printed PLA scaffolds significantly enhance the biological response of the human fetal osteoblast cell line (hFOB 1.19), guiding the in vitro differentiation for bone tissue regeneration.

## 2. Materials and Methods

### 2.1. Materials

Polycaprolactone (PCL) (80,000 g mol^−1^) was purchased from Sigma-Aldrich^®^, Gillingham, UK. Type A gelatin (50,000 g mol^−1^), obtained from pork skin, was acquired from MP Biomedicals^®^, LLC, Solon, OH, USA. Polylactic acid (PLA) (4060D, molecular weight = 188 g mol^−1^, 88% L-lactide and 12% D-lactide) was supplied by NatureWorks Ingeo^®^ Blair, NE, USA. In contrast, PLA filament (white, 1.75 ± 0.03 mm in diameter, printing temperature 190–210 °C) was purchased from KINGROON^®^ Shenzhen, China, and the glacial acetic acid (HAc) (≥99.7% of purity) was purchased from MEYER^®^ Vallejo, CA, USA.

### 2.2. Fabrication of Three-Dimensional PLA Scaffolds

The three-dimensional scaffolds (3D) were digitally designed with Rhinoceros Software (Rhino 8, 2024) (disks of 10 mm of diameter and 1 mm of height) and Ultimaker Cura Software (5.4.0) was used as a slicer for the printing pattern (zig-zag, layer height of 0.2 mm, printing line thickness of 0.4 mm, 20% infill and printing speed of 15 m s^−1^). A 3D-printer machine, Creality Ender 3-S1 (resolution of ±0.1 mm), was used to print the 3D disks layer-by-layer with a PLA filament (white, 1.75 ± 0.03 mm, KINGROOM^®^, Shenzhen Kingroon Tech Co., Limited, Hong Kong, China) at a temperature of extrusion of 200 °C, printing bed at 60 °C, and needle tip of 0.4 mm by FDM technique. The printed disks were disinfected by immersion in a 70% (*v*/*v*) ethanol solution for 30 min, then dried under vacuum at room temperature for 72 h. The samples were stored in a closed desiccator containing silica gel at room temperature and pressure conditions until use.

### 2.3. Scaffold Coating by Electrospun Nanofibers

The 3D-printed PLA disks were coated with different polymer nanofibers using different solutions and ES process configurations. Briefly, 0.6 g of Gt was dissolved in 5 mL of acetic acid (HAc) until dissolution to obtain a 12% (*w*/*v*) Gt solution. Likewise, 0.6 g of PCL and 0.6 g of PLA were separately dissolved in 4.5 mL of glacial acetic acid until a homogeneous solution formed. After being stirred for 24 h, 0.5 mL of deionized water was added 30 min before the ES process to obtain a 12% (*w*/*v*) PCL or PLA solution in HAc:H_2_O, 9:1 volume ratio [71]. After fixing the 3D-printed PLA disks onto the collector (aluminum foil, 5 × 5 cm), Gt, PCL, or PLA solutions were injected via a syringe pump (Inovenso Pump Systems, Istanbul, Turkey, model IPS 12S) through a single nozzle (20 G) at 0.5 mL h^−1^ to form monocomponent fibers. Alternatively, for the obtention of core/shell of PCL/Gt or PLA/Gt, the individual solutions were injected by two syringe pumps (Inovenso Pump Systems, model IPS 12S, KDS-100 syringe pump, KD Scientific, Holliston, MA, USA), connected to a coaxial needle (inner 25 G, outer 20 G) using PCL or PLA as inner solutions and Gt as the outer solution with flow rates of 0.2/0.5 mL h^−1^. All samples were obtained by applying 10 kV of voltage using a high-voltage power supply (SPELLMAN, Hauppauge, NY, USA, model RHR30PN30), imposing an electrode gap of 140 mm, at a controlled temperature (25 ± 5 °C) and relative humidity (35 ± 5%) (Table 1). The morphological properties of the fibers were investigated by an optical microscope (Olympus CX43, Olympus Corporation, Tokyo, Japan) equipped with a dark-field filter, and image acquisition was carried out via RisingView Software (macOS 2.1, 2023) (RisingTechnology, Boiling Springs, SC, USA, 2014). After fiber coatings, scaffolds were kept at room temperature in a desiccator under vacuum for 48 h to remove any residual organic solvent. Then, the scaffolds were sterilized under UV light in a laminar flow hood for 30 min on each side. The samples were placed in a sterile 24-well culture dish and sealed. The samples were stored in a closed desiccator containing silica gel at room temperature and pressure conditions until use.

### 2.4. Characterization of 3D-Printed PLA Scaffolds and Nanofiber Coating

A Binocular Stereoscopic Microscope (VELAB, Paris, France, VE-S7) was used to observe the printed disks at a magnification of 0.65×. Images were acquired using an integrated digital camera (VE-LX1400) and processed with VelabView Software^®^ (version x64, 2023).

To analyze the morphology of the nanofiber coating, a Scanning Electron Microscope (SEM) (JEOL-7800) at 2 kV in high-vacuum mode was used, by providing a gold sputter coating of the samples.

A High Resolution Transmission Electron Microscope (HR-TEM) (JEOL-ARM-200F) was used at 20 kV to analyze CNFs. SEM and TEM images were processed using DigitalMicrograph^®^ (Gatan Inc., Pleasanton, CA, USA, version 3.52.3932.0, 2022) and analyzed by ImageJ software (version 1.53t, 1.8.0_345-NIH, Bethesda, MD, USA), respectively. A representative sample of 200 fibers was used to measure the average fiber diameter. Images were converted to 8-bit, thresholded using Otsu’s method to segment pores from fibers. A total of 3000 interfibrillar pores were measured from micrographs for each group, with images taken randomly across the membrane surface to determine the pore area (μm^2^) and the percentage of pore area (%) [72]. The data were expressed as mean ± standard deviation (SD). Pore area (%) was calculated using Equation (1):

(1) Pore area (%) = (Total Pore Area)/(Total Image Area) × 100%.

Fourier-transform infrared spectroscopy (FT-IR) was performed using a Nicolet iS50R FT-IR spectrometer (Thermo Scientific, Waltham, MA, USA) equipped with an attenuated total reflectance (ATR) accessory. The spectra were registered from 4000 to 400 cm^−1^, with a resolution of 4 cm^−1^ and 32 scans per spectrum. Data acquisition was conducted using OMNIC software (version 9.5, 2015).

Thermogravimetric characterization was performed on a simultaneous thermogravimetric analyzer (STA-1200, Instrument Specialists Inc., Boerne, TX, USA). An amount of 20 mg of sample was used in an aluminum pan and evaluated over a temperature range from 25 °C to 600 °C at a heating rate of 10 °C min^−1^, under a nitrogen atmosphere with a flow rate of 30 mL min^−1^.

Water contact angle (WCA) of the samples was measured using a contact angle measuring system (VCA optima, AST Products, Inc., Billerica, MA, USA) equipped with a digital camera (CAM-Plus, ChemInstrument, Fairfield, OH, USA). For each measurement, a 5 μL droplet of deionized water was placed onto the surface of each sample. Every group was measured in triplicate. Notably, all the measurements were collected rapidly (about 1 s after the droplet deposition) to minimize the effect of the drop diffusion through the scaffold structure.

### 2.5. Biological Activity of 3D-Printed Scaffolds Coated with Nanofibers

The effects of the 3D-printed scaffolds coated with electrospun nanofibers on biocompatibility and bioactivity were assessed. Human fetal osteoblast cell line was purchased from ATCC (American Type Culture Collection, Manassas, VA, USA). hFOB cells were cultured in Dulbecco’s Modified Eagle’s Medium F-12 (DMEM F-12, Gibco, Grand Island, NY, USA) supplemented with 10% of fetal bovine serum (FBS) (Biosciences, Torrance, CA, USA) and 1% of antibiotic-antimycotic solution (streptomycin 100 μg/mL, penicillin 100 UI/mL and fungizone 0.3 μg/mL) (Sigma-Aldrich, St. Louis, MO, USA). For all experimental procedures, hFOB cells between passages 2 and 4 were used and incubated with 5% of CO_2_ at 37 °C and 100% relative humidity.

For the early cell adhesion test, the hFOB 1.19 cells were detached from the flask culture using 0.025% trypsin, and the scaffolds were seeded into 48-well plates at a density of 2 × 10^4^ cells per well. After 4 and 24 h of incubation, the scaffolds were washed three times with 1× phosphate-buffered saline (PBS) and fixed with 2% (*w*/*v*) glutaraldehyde (GTA) for 30 min at room temperature. After removing the GTA solutions, the scaffolds were washed three times with deionized water, and 0.2% (*w*/*v*) crystal violet solution was used to stain adherent cells. The samples were washed several times with deionized water to remove excess staining. The images of the top surface of scaffolds were acquired with an optical microscope (Olympus CX43) with an Industrial Digital Camera (RisingCam^®^, Fuzhou, China, U3CMOS).

Cell-Tracker™ (CMFDA, 5-chloromethylfluorescein diacetate) followed the cell adhesion test. Before seeding the cells, the cells were labeled with CMFDA at a 1:200 ratio using red phenol-free medium (RPMI 1×, Roswell Park Memorial Institute Medium, Corning, Mediatech Inc., Manassas, VA, USA), supplemented with 10% FBS and 1% antibiotic-antimycotic solution. After overnight cell recovery, the cells were detached from the flask using 0.025% trypsin, and 2 × 10^4^ cells were seeded onto the scaffolds. After 24, 48, or 72 h of incubation, cells were washed three times with PBS, then fixed with 2% GTA, followed by three washes with deionized water. The images of the top surface of the scaffolds were acquired with an epifluorescent microscope (AmScope, Irvine, CA, USA) with a Microscope Digital Camera (AmScope, MU1203-FL).

The cell viability test was performed by the MTT assay (3-[4,5-dimethylthiazol-2-yl]-2,5-diphenyltetrazolium bromide) (Invitrogen, Carlsbad, CA, USA; Thermo Fisher Scientific, Waltham, MA, USA). The scaffolds were seeded at 5 × 10^3^ cells/well in 24-well plates and incubated at 37 °C under 5% CO_2_ and 100% relative humidity. The complete media (supplemented with 2% FBS) was replaced every two days with fresh media until the measurement. After 1, 3, 7, 14, and 21 days of incubation, the media were replaced with 300 μL of fresh media containing 1:10 of 12 mM MTT solution. After 4 h of incubation, the media were removed, and the formazan crystals were dissolved in dimethyl sulfoxide (DMSO) for 15 min at room temperature under gentle agitation. 100 μL of solution was placed in a 96-well plate to measure the absorbance at 570 nm using a microplate reader (PKL PPC 142, PKL^®^ POKLER ITALIA, Salerno, Italy). The absorbance (A) is directly proportional to the number of viable cells and their metabolic activity. Cell viability (%) was calculated using Equation (2):

(2) Cell viability (%) = (A_sample_ − A_blank_)/(A_control_ − A_blank_) × 100%.

where A is the absorbance measured at 570 nm. The control group was set to 100% cell viability for each day’s measurement.

Mineralization was assessed using Alizarin Red S (ARS) staining. 5 × 10^3^ cells/well were seeded onto the scaffolds in DMEM-F12 complete medium for 24 h, then with fresh media. The media was replaced every two days with fresh media until the measurement. After 14 days of cultivation, the cell-seeded scaffolds were washed three times with 1× PBS and fixed with 2% (*w*/*v*) GTA solution for 30 min at room temperature. Then, the samples were rinsed three times with deionized water, and 500 μL of ARS 40 μM was added for 30 min to each sample. The samples were washed with DI water until no staining remained. The top surface of the samples was observed under an optical microscope (Olympus CX43) equipped with a dark-field filter, and digital images were acquired using the RisingView Software (RisingTechnology, 2014). For ARS quantification, the extraction was carried out by adding 250 μL of 10% (*v*/*v*) HAc at 85 °C for 10 min. Then, 100 μL of the sample solution was placed in a 96-well plate to measure the absorbance at 405 nm using a microplate reader (PKL PPC 142, PKL^®^ POKLER ITALIA).

Alkaline phosphatase (ALP) activity was determined using the ALP kit (Abcam ab83369, Cambridge, UK) according to the manufacturer’s protocol. The scaffolds were seeded with 5 × 10^3^ cells/well in osteogenic medium containing L-ascorbic acid (50 μg/mL), β-glycerolphosphate (10 mM), and dexamethasone (10 nM), supplemented with 10% FBS and 1% of antibiotic-antimycotic solution. The ALP assay was performed in culture after 3, 7, and 14 days. Briefly, the cell-seeded scaffolds were washed three times with PBS 1× and 250 μL of buffer lysis solution (0.2% of Triton X-100). For the assay, 80 μL of the lysate was transferred to a 96-well plate with 50 μL of 5 mM p-nitrophenyl phosphate (pNPP) at 37 °C for 60 min. Finally, the absorbance of the samples was measured in a microplate reader (PKL PPC 142, PKL^®^ POKLER ITALIA) at 405 nm, and the International Units (U/L) of ALP enzyme activity was calculated using a standard curve.

### 2.6. Statistical Analysis

The data were collected and analyzed in triplicate, expressed as the mean (SD), and analyzed using GraphPad Prism (version 10.6.1). One-way or two-way analysis of variance (ANOVA) was used for multiple groups, followed by Tukey’s post hoc test. Data were considered significantly different when *p* values were less than 0.05 (*p* < 0.05).

## 3. Results

### 3.1. 3D Scaffolds with Electrospun Fiber Coating

**Figure 1:**
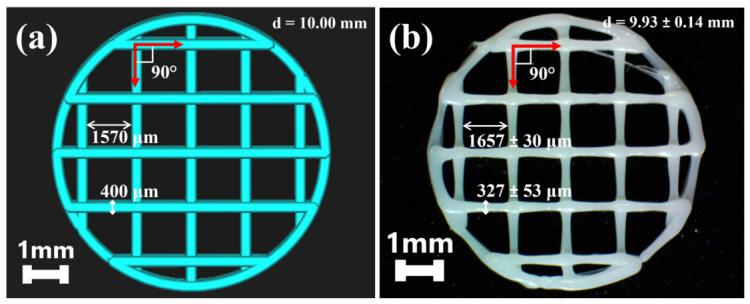
Comparison of the CAD/CAM model to the printed scaffold. (**a**) STL of 3D-printed disk (10 × 1 mm) designed into Rhino Software and slicer with Ultimaker Cura with 20% of infill density, and (**b**) 3D-printed disk with PLA filament at 200 °C and speed of 15 mm/s with zig-zag infill by Fused Deposition Modeling (FDM). Binocular Stereoscopic Microscope, VELAB, VE-S7, 0.65×.

3D scaffolds were designed via Rhino Software (version 8) and sliced by Ultimaker Cura Software, collecting alternated vertical and horizontal strands (Figure 1a). After the printing process, 3D disks (Figure 1b) showed 9.93 ± 0.14 mm as their diameter, exhibiting a 90° array and an in-phase printing pattern, and a single line thickness of 327 ± 53 µm. The infill density was equal to 20%—to theoretically obtain a scaffold with 80% porosity that mimics the trabecular bone porosity, while the pore size equal to 1657 ± 30 μm. The experimentally measured dimensions of the 3D-printed disks closely matched the geometrical parameters of the designed model (i.e., 10 mm of diameter with line thickness of 400 µm and pore sizes of 1570 µm). Differences between theoretical and experimental data were attributed to the effect of the punctual junctions along the filaments and to the FDM resolution variations—equal to ±0.1 mm.

**Figure 2:**
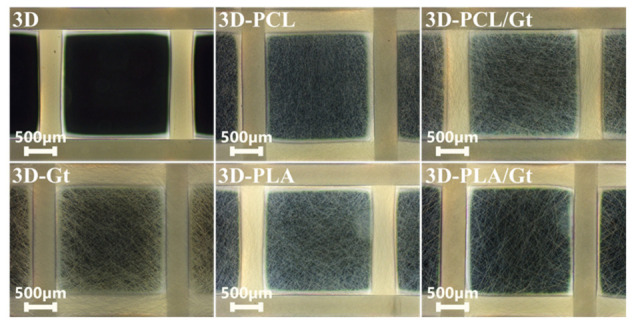
3D-printed PLA scaffolds, uncoated and coated with electrospun nanofibers of Gt, PCL, PLA, and CNFs of PCL/Gt and PLA/Gt. Dark-field microscopy (DMF), Olympus CX43, 4×.

3D-printed disks were coated by nannofibers produced via ES, a highly versatile technique suitable to impart changes in topography and chemical composition of the surface, independently on the structural complexity of the substrate below. Dark-Field Microscopy (DFM) images show the surface morphology of the 3D scaffolds after the deposition of the nanofibrous coatings collected via single-nozzle (Gt, PCL, and PLA) and CNFs via coaxial-nozzle (PCL/Gt and PLA/Gt) (Figure 2).

### 3.2. Morphological and Physico-Chemical Characterization

**Figure 3:**
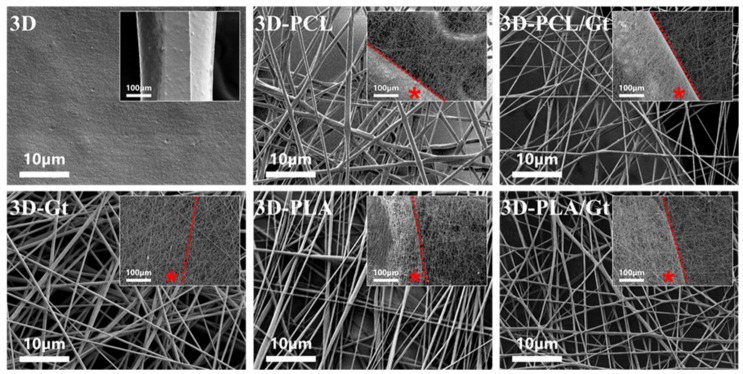
3D-printed PLA scaffolds, uncoated and coated with electrospun nanofibers of Gt, PCL, PLA, and CNFs of PCL/Gt and PLA/Gt (red asterisk indicates the 3D-printed line zone). Scanning Electron Microscopy (SEM), SEI, 2500×. Inset images, 250×.

A comparative analysis of morphological properties of different nanofibers was performed via SEM (Figure 3). As a function of the experimental setup used and composition of the solution used, different characteristic sizes of nanofibers were recognized. In the case of fibers processed via single-nozzle, average diameters of 496 ± 128 nm, 375 ± 124 nm, and 483 ± 199 nm were calculated for Gt, PCL, and PLA, respectively. Otherwise, a slight reduction of fiber diameters to 287 ± 74 nm and 316 ± 124 nm was recorded in the case of coaxial fibers, PCL/Gt and PLA/Gt, respectively (Supplementary Materials Figure S1 and Table S1). This experimental evidence can be ascribed to the stretching effect exerted by the electric field on polar macromolecules of Gt along the outer shell. This interaction induces a high charge density at the interface with the under phase, promoting the formation of thinner fibers with respect to PCL or PLA alone [73,74].

Accordingly, a reduction in pore sizes was recognized, mainly in the case of random fiber distribution. Pore area analysis (Supplementary Materials Table S1) showed a decrease in pore sizes in the case of Gt added fibers. In more detail, pore area decreases from 2.67 ± 1.83 μm^2^ and 1.89 ± 0.81 μm^2^ in the case of PCL and PCL/Gt, and from 2.39 ± 1.51 μm^2^ to 1.41 ± 0.47 μm^2^ in the case of PLA and PLA/Gt, respectively. In contrast, the percentage of total pore area showed an opposite trend. In particular, it increased from 22.10 ± 5.67% to 48.54 ± 5.59% for PCL and PCL/Gt fibers, and from 37.06 ± 6.43% to 46.72 ± 5.97% for PLA and PLA/Gt, respectively.

**Figure 4:**
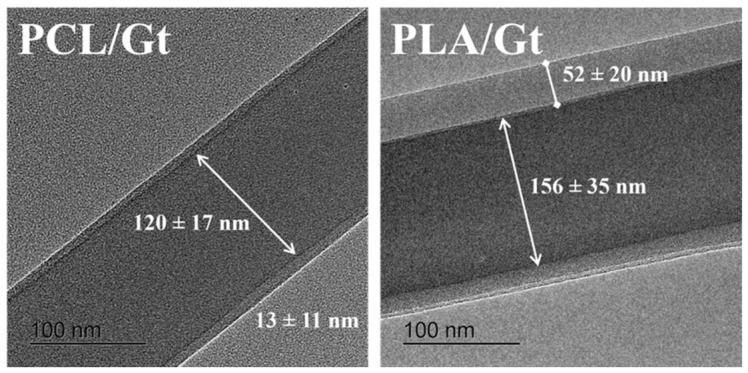
Core/shell structure of CNFs of PCL/Gt and PLA/Gt. High-resolution transmission electron microscopy (HR-TEM), SEI, 100,000×.

Furthermore, the morphology of CNFs was investigated via TEM to evaluate their peculiar inner structure. TEM images of PCL/Gt and PLA/Gt nanofibers were reported in Figure 4. They showed a clear core/shell structure where darker areas were related to PCL or PLA core, while brighter ones to the Gt-based shell. Quantitative measurements performed on selected images highlighted the characteristic sizes of CNFs—i.e., core diameter of 120 ± 17 nm and 156 ± 35 nm and shell thickness equal to 13 ± 11 nm and 52 ± 20 nm—for PCL/Gt and PLA/Gt, respectively. Noteworthily, an evident increase in the shell thickness was recognized in the case of PLA/Gt with respect to PCL/Gt CNFs, with equal flow rate ratios applied, related to the different interface interaction of the used solutions, due to their different hydrophobic properties.

An accurate investigation was preliminarily assessed to evaluate the effect of voltage on the formation of core/shell structures (Supplementary Materials Figure S2). Independently of the applied voltage (from 8 to 12 kV), the characteristic sizes of PCL/Gt CNFs did not significantly change. At 8 kV, the injection flow was unstable, resulting in only a few fibers being deposited. At 12 kV, instability phenomena preferentially occurred, and only a limited number of core/shell fibers were observed. On the other hand, in the case of PLA/Gt, at 8 kV, mostly individual fibers formed, whereas at 12 kV, fiber formation was irregular, with cores asymmetrically displaced into the external shell, probably due to jet instability. Based on these experimental results, a 10 kV voltage was used to fabricate CNFs as a scaffold coating for biological studies.

**Figure 5:**
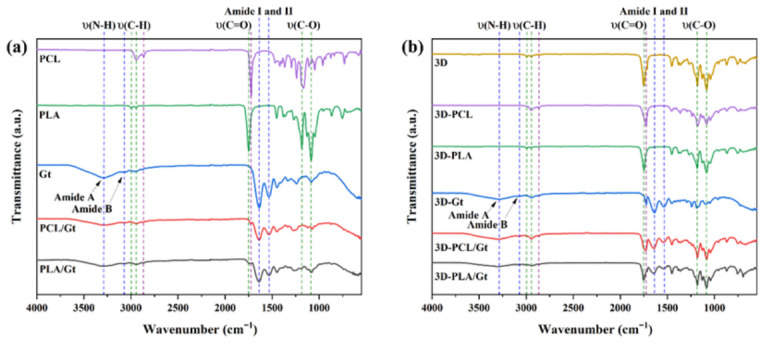
FTIR-ATR spectra for (**a**) electrospun nanofibers used as coatings and (**b**) 3D-printed PLA scaffolds coated with nanofibers.

Infrared spectra of the nanofibers used for the coatings were collected to investigate the effect of process conditions on the characteristic signals of the polymers (Figure 5). In the IR spectrum of PCL, the following bands are observed: the asymmetric and symmetric stretching vibrations of the C-H bonds (from the CH and CH_2_ groups) at 2943 and 2865 cm^−1^, respectively, the C=O stretching bond (from the carbonyl group) at 1722 cm^−1^, and C-O stretching (from the ester group) at 1165 and 1047 cm^−1^ [41,75]. The PLA spectrum shows bands associated with the asymmetric and symmetric stretching vibrations of the C-H bonds (from the CH and CH_2_ groups) localized at 2994 and 2944 cm^−1^, the C=O stretching bond (from the carbonyl group) at 1748 cm^−1^, and C-O stretching (from the ester group) at 1182 and 1084 cm^−1^ [7,76]. For Gt, signals appeared due to the N-H stretching bond (from the amide A group) at 3291 cm^−1^, the C-H stretching vibration (from amide B) at 3076 cm^−1^, the C=O stretching (from the carbonyl group of amide I) at 1635 cm^−1^; the C-N stretching bond (from amide II) at 1537 cm^−1^, and C-O stretching at 1161 and 1081 cm^−1^ [41,57,75,76,77,78,79]. These signals are representative of pure polymers.

In the case of CNFs, a combination of signals from polyesters-Gt was observed. In particular, a red shift of the IR band (from 3291 to 3283 cm^−1^), associated with amide A, was observed in CNFs, indicating a lower vibrational energy. This shift suggests an interaction between Gt and polyesters via hydrogen bonding [79]. Additionally, an increase in the intensity of the bands corresponding to C=O (carbonyl) at 1750 cm^−1^ and C-O (ester) at 1180 cm^−1^ and 1080 cm^−1^, was noted. This enhancement can be attributed to a higher PLA content in the 3D-printed scaffold [80].

No solvent signals were observed (HAc or water), indicating that all solvent residues were eliminated after 72 h of vacuum.

**Figure 6:**
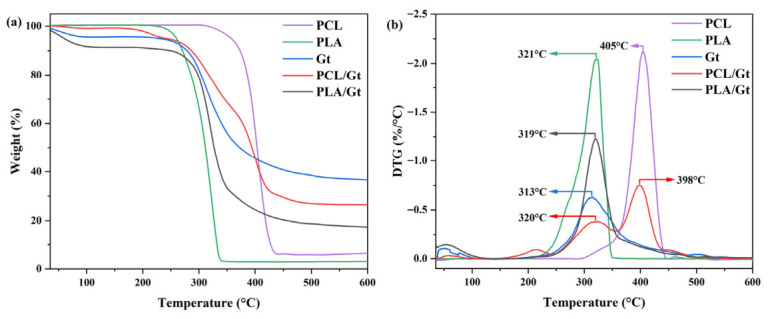
(**a**) Thermogravimetrical analysis and (**b**) derivative (TGA and DTG) for nanofibers of Gt, PCL, PLA, and coaxial electrospun nanofibers PCL/Gt and PLA/Gt.

Thermogravimetric analyses were also performed on the nanofibrous coatings to further investigate the effect of process conditions on scaffold thermal stability (Figure 6).

Figure 6a showed that polymer degradation takes place for PCL, PLA, and Gt by a single step, with a characteristic degradation temperature (Td) of 405 °C for PCL, 318 °C for PLA, and 313 °C for Gt, as confirmed by the maximum value of the derivative curves (in the square of Figure 6). In the case of CNFs, degradation should occur in two steps, reflecting the presence of two distinct components. This is evident in the case of PCL/Gt, where the first peak at 320 °C corresponds to near-pure Gt decomposition, while the second peak at 397 °C is related to PCL. In the case of PLA/Gt, the Td of PLA and Gt are very close so that an overlap of the decomposition peaks can be recognized with an average Td equal to 319 °C (see Supplementary Data Table S2). Notably, a characteristic peak around 100 °C was observed for all the samples, related to physical water desorption, except for PCL-Gt, where the temperature increase was lower than 200 °C.

**Figure 7:**
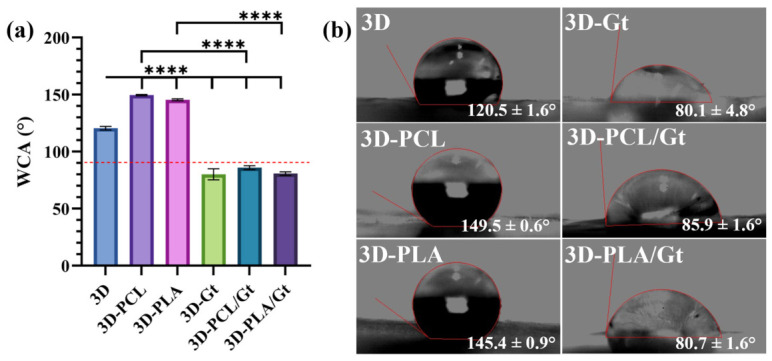
Water contact angle (WCA) for the top surface of the 3D-printed PLA scaffolds coated with nanofibers: (**a**) Quantitative comparison of WCA measurements (*n* = 3). WCA < 90° indicates a hydrophobic surface (dashed red line). One-way ANOVA and Tukey’s post hoc test (**** *p* < 0.0001). (**b**) Droplet shape images for WCA measurements on the top surface of scaffolds.

WCA measurements were obtained to study the effect of NF coatings on the surface wettability of the 3D-printed scaffolds. Results in Figure 7a evidence that the addition of PCL or PLA nanofibers tended to increase the hydrophobicity of the 3D scaffolds. The uncoated 3D scaffold showed a contact angle of 120.5 ± 1.6°; in the presence of PCL and PLA nanofiber coatings, these values significantly increased to 149.5 ± 0.6° and 145.4 ± 0.9° (*p* < 0.0001), respectively. Otherwise, for the Gt nanofiber coating, as expected, the surface shifted to a hydrophilic behavior compared with the uncoated 3D scaffold to 80.1 ± 4.8° (*p* < 0.0001). In addition, these hydrophilic properties are also observed for CNF coatings. A contact angle of 85.9 ± 1.6° for PCL/Gt and 80.7 ± 1.6° for PLA/Gt was observed. These values are comparable with those of the pure Gt coatings and gave statistically different results from those of pure PCL- or PLA-coated or uncoated scaffolds (*p* < 0.0001). Qualitatively, water droplets showed a more rounded shape in the case of polyester-based scaffolds, while tended to be flattened in the case of Gt-coated surfaces, confirming their positive contribution in terms of wettability. Accordingly, In the case of Gt-based coatings the droplet tended to form a contact angle below 90° (see the dashed red line), while in the case of polyesters, given their intrinsic hydrophobic nature, it increased by around 25–30°, thus confirming a reduction in the surface wettability. Hence, it is possible to conclude that the surface modification of 3D scaffolds via fibrous coatings allows for the modulation of the wettability capacity of the printed scaffolds, to some extent, depending on the nature of the polymers used.

### 3.3. In Vitro Response

**Figure 8:**
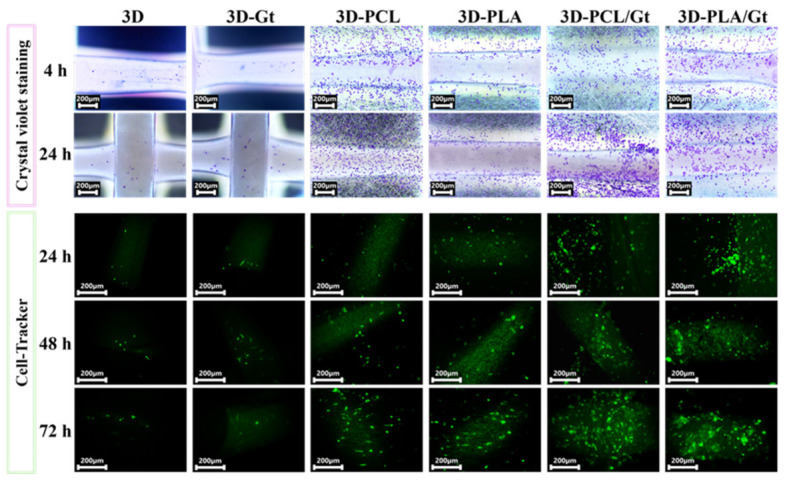
Adhesion test images for hFOB 1.19 cell line on the top surface of the 3D-printed PLA scaffolds coated with nanofibers by crystal-violet staining (CVS) at 4 and 24 h. Dark-field microscopy (DFM), Olympus CX43 optical microscope, 20×. Fluoresce adhesion test at 24, 48, and 72 h followed by Cell-Tracker™ (CMFDA, 5-chloromethylfluoresceindiacetate). Fluorescence microscope, AmScope, 10×.

The cell adhesion tests show that enhanced attachment was observed on 3D-printed PLA scaffolds coated with nanofibers (Figure 8). The adhesion tests, followed by crystal violet staining, showed that modifying 3D-printed PLA scaffolds with nanofibers and CNFs increased cell anchoring capacity from the first 4 h. The highest surface coverage by cell density is observed for Gt-based nanofibers, with increased cell density in PCL/Gt and PLA/Gt at 24 h compared with the controls (pure polyester and Gt fiber compositions, as well as uncoated 3D-printed PLA scaffolds). The fluorescence assay showed that cells preferred to retain and adhere to CNFs during the first 24 h. However, a greater surface coverage of viable cells is observed by fluorescence at 72 h in PCL/Gt and PLA/Gt, both in the printed lines and in the pore areas. These results demonstrate that the surface modification of 3D-printed PLA scaffolds with coating nanostructures, such as ES-derived fibers, enhances early cell adhesion. This phenomenon may positively impact viability and differentiation assays, suggesting that combining these technologies could significantly improve outcomes in tissue engineering and cell regeneration applications.

**Figure 9:**
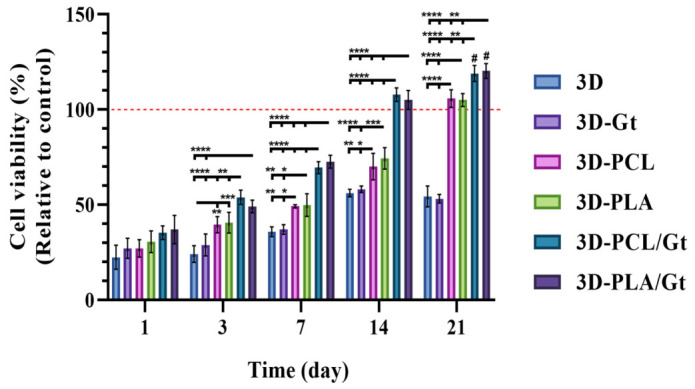
Cell viability (%) for the hFOB 1.19 cell line cultivated on the 3D-printed PLA scaffolds, uncoated and coated with nanofibers. Two-way ANOVA and Tukey’s post hoc test. * *p* < 0.05, ** *p* < 0.01, *** *p* < 0.001 and **** *p* < 0.0001. # *p* < 0.0001 vs. the control. The dashed red line represents the control group, taken as 100% cell viability for each day.

Cell viability tests for hFOB cells in culture showed improved viability with 3D-printed PLA scaffolds coated with fibrous material (Figure 9). On day 1 of culture, no differences were observed between the evaluated groups. However, on day 3, the 3D and 3D-Gt scaffolds showed lower viability, 35.87 ± 2.54% and 37.05 ± 2.49%, respectively. Cell viability of 3D was lower compared with the scaffolds coated with PCL (*p* < 0.01) and PLA (*p* < 0.001) fibers, which showed 49.28 ± 0.82% and 49.84 ± 5.96%, respectively. In addition, the differences were higher than those for the 3D-PCL/Gt (*p* < 0.0001) and 3D-PLA/Gt (*p* < 0.0001) scaffolds, with 53.89 ± 3.74% for 3D-PCL/Gt and 49.17 ± 3.32% for 3D-PLA/Gt. On day 7, an increase in cell viability was observed for the CNF coatings. The cell viability for 3D-PCL/Gt was 69.57 ± 3.17% and for 3D-PLA/Gt was 72.65 ± 3.36%, showing the main differences compared to the 3D with 35.87 ± 2.54%, 3D-Gt with 37.05 ± 2.49%, and 3D-PCL with 49.28 ± 0.82% scaffolds (*p* < 0.0001). The 3D and 3D-Gt have lower cell viability than 3D-PLA (49.84 ± 5.96%), and this difference is also observed compared with 3D-PCL (*p* < 0.01 compared with 3D and *p* < 0.05 compared with 3D-Gt). On day 14 of culture, the 3D-PCL/Gt and 3D-PLA/Gt groups show 107.83 ± 3.57% and 105.08 ± 4.95%, respectively, and exhibit the greatest differences compared to the other groups (*p* < 0.0001). The 3D and 3D-Gt scaffolds had the lowest viability. The 3D scaffold had 56.17 ± 2.00% viability and differed significantly from 3D-PCL with 70.13 ± 6.90 (*p* < 0.01) and 3D-PLA with 74.41 ± 5.63% (*p* < 0.0001), while 3D-Gt had 58.20 ± 1.78% and was different from 3D-PCL (*p* < 0.05) and 3D-PLA (*p* < 0.001). Finally, on day 21 of culture, the 3D-PCL/Gt and PLA/Gt scaffolds had a viability of 118.98 ± 4.21% and 120.30 ± 3.87%, respectively. This represents the higher values of cell viability and showed significant differences (*p* < 0.0001), compared to the control (defined as 100% viability, dashed red line), 3D (54.44 ± 5.52%), 3D-Gt (53.17 ± 2.26%); while showing lower differences (*p* < 0.01) compared to 3D-PCL (105.81 ± 4.68%) and 3D-PLA (105.07 ± 3.42%). The 3D-PCL and 3D-PLA scaffolds showed differences (*p* < 0.0001) compared with the 3D and 3D-Gt scaffolds.

These results are consistent on days 7, 14, and 21 of cell culture and may correlate with cell adhesion, suggesting that enhanced cellular attachment may have facilitated the rapid establishment of hFOB populations and, consequently, a more efficient proliferation process. These findings indicate that the surface modification of 3D-printed PLA scaffolds with coating nanofibers and CNFs enhances cell viability.

**Figure 10:**
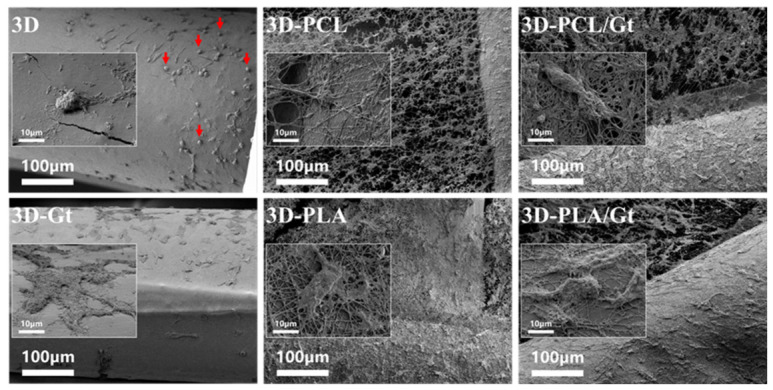
3D-printed PLA scaffolds coated with nanofibers after 14 days of culture with hFOB 1.19 cell line: 3D-uncoated, nanofibers of Gt, PCL, PLA, and CNFs of PCL/Gt and PLA/Gt. SEM, SEI, 250×. Inset images from 2500×. Red arrows point to the rounded cells from the uncoated scaffold.

SEM images of cultured hFOB 1.19 cells on day 14 showed different osteoblast morphologies (Figure 10). For the 3D-printed PLA scaffolds, cells appeared more rounded than in the other samples. This behavior was also observed for the PCL and PLA coatings; however, a higher cell density was evidenced. On the other hand, the 3D-printed PLA scaffolds with Gt-based single-nozzle, and CNFs showed the most spreading, star-shaped morphology of osteoblasts, indicating that Gt helps cell attachment because more focal adhesion sites are available. Also, the extracellular and mineral matrix formation can be observed for these samples. The nanofibrous structures remained attached to the 3D-printed PLA scaffolds after 14 days of culture, despite exhibiting swelling, without requiring post-processing following the physical surface coating.

**Figure 11:**
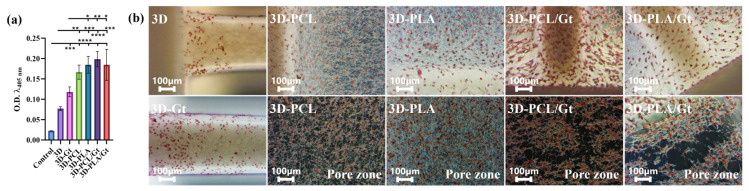
ARS staining and measurement for 3D-printed PLA scaffolds coated with nanofibers of Gt, PCL, and PLA, and CNFs of PCL/Gt and PLA/Gt after 14 days of cell culture. (**a**) Comparison of ARS extracted from scaffolds. One-way ANOVA and Tukey’s post hoc test. * *p* < 0.05, ** *p* < 0.01, *** *p* < 0.001, **** *p* < 0.0001. (**b**) Dark-field microscopy (DFM) images of the top surface of scaffolds: Olympus CX43 optical microscope, 20×.

ARS staining shows high mineral deposition for the 3D-printed PLA scaffolds coated with nanofibers on day 14 (Figure 11). Figure 11a shows the extracted ARS quantification for the scaffolds. The 3D-PCL/Gt scaffolds had the highest O.D. value (0.198 ± 0.019), whereas the control group had the lowest O.D. value (0.024 ± 0.0004). The main differences between the control group and all groups are statistically significant (*p* < 0.0001), (*p* < 0.001, for 3D and 3D-Gt). The O.D. for 3D (0.077 ± 0.005) was lower compared to the 3D-PCL (0.167 ± 0.017) (*p* < 0.01); 3D-PLA (0.185 ± 0.021) (*p* < 0.001), 3D-PCL/Gt (*p* < 0.0001), and 3D-PLA/Gt (0.185 ± 0.038) (*p* < 0.001). Meanwhile, the 3D-Gt (0.118 ± 0.012) showed differences compared to the 3D-PLA and 3D-PLA/Gt (*p* < 0.05), and higher differences with 3D-PCL/Gt (*p* < 0.01). These results are consistent with the images in Figure 11b, which show that 3D-PCL/Gt exhibits greater calcium deposition along the printing line and within the 3D-printed pore zone, indicating that the combination of micro- and nanostructures in 3D-PCL/Gt optimizes this phenomenon.

This observation relates to early cell adhesion and proliferation, followed by osteoblast maturation and mineral matrix deposition, indicating that fibrous coatings play an osteoconductive role by modifying the surface of 3D prints.

**Figure 12:**
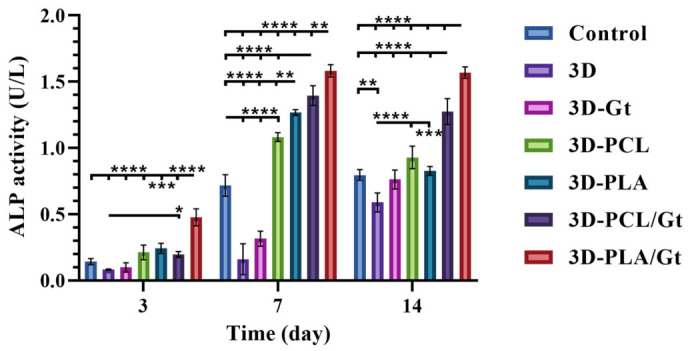
ALP assay for 3D-printed PLA scaffolds coated with nanofibers of Gt, PCL, and PLA, and CNFs of PCL/Gt and PLA/Gt after 3, 7, and 14 days of cell culture. Two-way ANOVA and Tukey’s post hoc test. * *p* < 0.05, ** *p* < 0.01, *** *p* < 0.001, **** *p* < 0.0001.

To study the osteogenic behavior of hFOB 1.19, the ALP enzymatic activity (U/L) was measured. The results in Figure 12 showed greater ALP activity on day 3 for 3D-PLA/Gt (0.47 ± 0.06) compared to 3D-PLA (0.24 ± 0.04) (*p* < 0.001). Also compared with the other groups (*p* < 0.0001), 3D (0.08 ± 0.004), 3D-Gt (0.01 ± 0.03), 3D-PCL (0.21 ± 0.05), including the control (0.14 ± 0.02), indicating early activity for these scaffolds. The highest ALP activity was observed on day 7 for both PCL/Gt (1.39 ± 0.07) and PLA/Gt (1.58 ± 0.04). For PLA/Gt, activity was higher compared to PCL/Gt (*p* < 0.01) and was also higher compared to the other groups (*p* < 0.0001); control (0.72 ± 0.08), 3D (0.16 ± 0.12), Gt (0.32 ± 0.06), PCL (1.08 ± 0.03), and 3D-PLA (1.27 ± 0.021). PCL/Gt activity was higher (*p* < 0.0001) than 3D, 3D-Gt, and PCL and the control groups. Finally, on day 14, the persistence of high activity for PLA/Gt (1.57 ± 0.04) and subsequently for PCL/Gt (1.27 ± 0.09) became evident, being significantly different (*p* < 0.0001) compared with control (0.79 ± 0.04), 3D (0.59 ± 0.07), Gt (0.76 ± 0.07), PCL (0.93 ± 0.08) and 3D-PLA (0.83 ± 0.03). In contrast, 3D scaffolds showed the lowest ALP activity compared with the control (*p* < 0.01), 3D-Gt (*p* < 0.05), 3D-PCL (*p* < 0.0001), and 3D-PLA (*p* < 0.001). For the other groups, there were no differences in ALP activity compared to the control group.

These results suggest early ALP activity, with the highest activity on day 7 for scaffolds with coaxial coatings, such as PCL/Gt and PLA/Gt. PLA/Gt exhibited enhanced ALP activity, suggesting that it more effectively promotes the early stages of hFOB osteodifferentiation, ultimately leading to mineralized tissue formation. This strongly correlates with previous studies on cell adhesion and proliferation, demonstrating that the use of Gt-based nanofiber coatings enhances the biological performance of 3D scaffolds through surface modification and represents promising results for BTE applications.

## 4. Discussion

In the last twenty years, 3D-printing technologies have been largely used to fabricate 3D scaffolds for bone regeneration, showing several benefits in terms of in vitro cell interaction, and some constraints mainly due to limited control of surface properties at the sub-microscale [34]. In order to enhance the cell interaction, an interesting route is to impart specific surface properties through chemical/physical cues able to influence cell interactions by triggering cell-linkage sites related to new bone formation [81,82,83].

Previous studies indicated that 3D scaffolds with highly ordered architectures and average pore sizes around 300–350 μm are optimal for in vitro studies [84,85]. Other works remarked that interconnected pores with sizes ranging from (0.1–10 μm) efficiently support fluid and molecular transport with benefits in the osteoinduction process [86], while bigger pores—over 300 μm in diameter—are crucial to promote proliferation and in vivo cell osteointegration [87] as well as new blood-vessel formation [88]. In particular, it is recognized that smaller pores, ranging from 100 to 200 μm, are preferred by osteoblasts after implantation for conducting the mineralization process [89]. In this view, the peculiar architecture (i.e., a theoretical 80% porosity) of 3D-printed scaffolds mimics the natural structure of cancellous (trabecular) bone, characterized by voids with a porosity range of 50% to 90% of the tissue volume [88,90,91]. More recent studies have also remarked the role of pore size distribution on the regeneration processes of differently dense bone tissues (cortical/cancellous), demonstrating how pore-size-graded scaffolds, with smaller pores—about 400 µm—on the periphery and bigger ones—up to 1000 µm—in the center, support a more efficient mass transport and increased osteogenesis and vascularization in comparison with structures with uniform pore sizes [92].

Herein, it was proposed to customize the scaffold surface by coating electrospun nanofibers. Different compositions and ES setup configurations were investigated to validate the use of Gt-based core/shell fibers as optimal strategy to coat 3D-printed PLA scaffolds. Despite being poorly investigated in the literature [37,92,93], nanofiber coatings on 3D-printed scaffolds offer an easy yet effective strategy to enhance scaffold interface properties, particularly for bone regeneration.

First, the deposition of electrospun nanofibers onto the 3D-printed PLA scaffold surface allows for significantly reduced void size available to cells. Secondly, the deposition of core/shell nanofibers with a Gt outer shell exposes recognized biochemical signals to cells. The reduction in fiber diameters in PCL/Gt and PLA/Gt coatings is related to the Gt content, mainly due to the reduction in the viscosity when the interface of core/shell fibers is forming because the lower molecular weight of Gt compared with PCL or PLA causes a decrease in the solution viscosity in the jet ejection [73]. In this context, the high charge density characteristic of Gt solutions contributes to this phenomenon [74].

Previous studies reported the preparation of core/shell nanofibers (i.e., PCL/Gt or PLA/Gt) using non-green solvents, such as trifluoroethanol (TFE), hexafluoroisopropanol (HFP), chloroform, and dimethylformamide (DMF) [18,41,43,59,69,78,91,94,95,96], with adverse effects on cell interactions. Contrariwise, here it is proposed, as a first step, the fabrication of PCL/Gt and PLA/Gt coaxial (core/shell) electrospun nanofibers using an eco-friendly, less toxic solvent mixture based on water and acetic acid, with relevant implications for biological response. In the recent past, other workers have proposed the use of glacial acetic acid alone [97] or in combination with DCM [98] to form green electrospun fibers. According to the infrared spectra, no modification of the characteristic peaks was observed in the case of pure polymer formulations [34,57,75,99,100], nor was any signal ascribable to traces of solvent used recorded after the ES process. Moreover, the presence of Gt at the outer shell of the fibers did not influence the decomposition behavior of constituent polymers, as reported by thermogravimetric analysis [14,41], but it increased the fibers’ surface hydrophilicity, as confirmed by the decrease in WCA in the presence of the nanofiber coating, being able to improve the cell interface [101]. Similar behaviors were documented in several studies using Gt in combination with hydrophobic/semi-hydrophobic polymers [21,74,90,102,103]. Here, this effect is also corroborated by the contribution of pores in the electrospun coating, providing an increase in WCA per se from 120° to 145°/150° compared with uncoated 3D-printed PLA scaffolds [72,104].

Starting from this experimental evidence, the effect of nanofiber coating on the biological response is examined. According to previous in vitro studies [105,106,107], herein, different biological assays were conducted to validate the biocompatibility (adhesion and proliferation) and differentiation (mineralization and ALP activity) of the hFOB 1.19 cell line. The adhesion test evidenced enhanced cell attachment on the top surface of the scaffolds at 24 h for Gt-based CNF coatings. After 72 h, it became clear that the early stages of adhesion were established by the surface’s hydrophilicity [41]. The proliferation assay showed that after day 3, the cultivated cells prefer the coated surfaces, as well as in the adhesion test, followed by an increment of viability at days 7, 14, and 21 for Gt-based CNF coatings onto the 3D-printed PLA scaffolds; 21-day significance differences were observed for this against the control, taken as 100%.

The results of the adhesion and viability tests can be explained by the ease of hydration with culture media, and the cell–scaffold interaction occurs more quickly than with uncoated scaffolds. Once the attachment was established, the subsequent cell growth step took place. A greater difference for the scaffolds coated with CNFs, both PCL/Gt and PLA/Gt. This behavior has been reported in some studies combining polymer-based biomaterial with collagen or Gt [67,68,69,108,109,110,111,112].

It is known that some Gt’s may promote cell attachment via integrins, as they contain an RGD sequence that facilitates this interaction. For this reason, Gt—as well as collagen—has been used for scaffold development due to its molecular recognition mechanism [4,113]. The principal disadvantage of Gt, including collagen, is its high water solubility. Consequently, other research has employed cross-linking methods to prevent leaching into the media [114,115,116]. However, as the Gt dissolves, the cells could establish early focal adhesions with the polyester matrix to continue the proliferation process and osteo-matrix generation within a cross-linking method before the culture. Once the attachment was established, the subsequent cell growth step took place.

SEM images of the cultivated 3D-printed PLA scaffolds can be correlated with the previously mentioned observations.

On day 14, matrix generation around the cells is evident, with a preference for the cells to adopt star-shaped morphologies and membrane projections that help anchor the scaffold surface [16]. This behavior may be induced by topographical signals from the nanofibrous texture and by biochemical signals from the presence of Gt, in agreement with previous studies on bicomponent fibers [117,118]. In contrast, rounded cells can be observed on uncoated surfaces, which can be explained by the hydrophobic nature of PLA [110] and by pore sizes exceeding the scaffold dimension manufactured via FDM deposition [34].

Thin nanofiber layers are fragile upon contact and can be intentionally detached, as well as by conventional electrospun membranes with multiple stacked layers. Since these are physical adhesives, they must be handled carefully to avoid puncturing the electrospun thin layers. However, at the end of the in vitro culture study after 14 days (Figure 10), the SEM images showed an adhesion stability of the fibers attached to the surface of the 3D-printed materials, maintaining their integrity, this adhesion fibers-substrate behavior was also observed by Rosales-Ibáñez, R. et al. in 2023 [119] without the use of an organic solvent-based adhesive, such as dichloromethane [120]. Traces of solvent during deposition may contribute to the adhesion of the first layer of fibers in this interface formation. In the case of Gt nanofibers, these are lost due to their solubility in aqueous media. It was also expected that the shells of coaxial nanofibers would be lost during this period. However, the integrity of the fiber cores (PCL or PLA) remains, and the Gt has already fulfilled its function by promoting cell adhesion.

Calcium mineral deposits have been linked to the osteogenesis process due to cell maturation to form mineralized bone tissue [121]. The qualitative Alizarin Red S (ARS) staining test showed a higher accumulation of calcium deposits on day 14 on the top surfaces of 3D-PCL/Gt and 3D-PLA/Gt compared with the 3D uncoated scaffolds, which was confirmed by the quantification of the extracted ARS (*p* < 0.0001), suggesting that a maturation of pre-osteoblasts to a mineralizing phenotype occurs to further bone matrix formation. This behavior was observed on all surfaces around the printed lines and in the pore areas, with a preference for osteo-matrix deposition on the PCL/Gt coatings. It is important to note that osteogenic inducers were not required for this assay, highlighting the possibility of relying on osteoconductive properties arising from topographic changes [122] upregulated by the nanofibrillar coatings, in addition to the Gt incorporation that enhances the cell attachment to the surfaces with moderate wettability properties [57,123,124]. In addition, fiber coating diameters of Gt-based CNFs around 300 nm could enable higher cell–material interactions, as demonstrated by cell attachment, indicating that the surface wettability (WCA < 90°) and the fiber coating size contribute synergistically to osteoconductive properties that promote osteogenesis.

ALP is a biological enzyme that serves as a marker for early stages of osteoblast differentiation and is expressed in bone-forming cells that are directly involved in early osteogenesis due to its ability to promote cell maturation and subsequent mineralized tissue generation [121,125,126,127]. The ALP enzymatic activity results showed that the maximum activity for the PLA/Gt coatings occurred on day 7 (1.58 ± 0.04 U/L), which was maintained until day 14 (1.57 ± 0.04 U/L) of cultivation, compared with other groups (*p* < 0.0001). Since day 3, early osteodifferentiation was evident, followed by the maximum activity of PCL/Gt coatings at day 7 (1.39 ± 0.07 U/L), which presented a decrease on day 14 (1.27 ± 0.09 U/L), which could be related to the maturation of osteoblast cells to start forming a mineralized matrix. These findings are related to the results observed in ARS staining, indicating that the calcium deposits formed after high levels of ALP were reached. This suggests that the CNF coatings play an important role in upregulating osteodifferentiation by surface-modifying the topographic and wettability properties of 3D prints [122]. In contrast, enhanced osteogenic differentiation was reported for PLA-containing nanofibrous scaffolds compared to pure PCL, mainly attributed to the increased scaffold stiffness with increasing PLA content; however, they used a 3D-nanofibrous scaffold of PCL/PLA [55], which implicates that Gt content could play a critical role in modulating this approach through surface wettability modifications, as it was described to stimulate cell adhesion [128]. It should also not be overlooked that Gt enhances these properties, as it has been shown to affect bone regeneration both in vitro and in vivo [129,130,131].

Therefore, the proposed study demonstrated the effectiveness of nanofibrous coatings on 3D-printed PLA scaffolds, as a tool to improve physicochemical properties (i.e., wettability) that affect the in vitro response at the interface with hFOB cells. However, several aspects need to be explored in more detail in order to implement 3D-printed PLA scaffolds suitable for bone implants. In order to more faithfully reproduce the hierarchal organization of a bone-like architecture, the implementation of tailored layer-by-layer strategies by the use of integrated additive manufacturing approaches should be investigated to combine/alternate 3D-printed and electrospun layers along a predefined thickness [132]. In this context, the use of 3D structural models with different pore sizes could help to optimize the suitable structural parameters to promote cell adhesion, proliferation, and osteoblastic differentiation in the presence of electrospun fibers. Herein, only initial evidence of the scaffold capability to support ALP activity and mineral phase deposition was proposed. Further investigations for the quantification of osteogenic markers expression (e.g., osteopontin (*OPN*), osteocalcin (*OCN*), runt-related transcription factor 2 (*RUNX2*), and bone sialoprotein (*BSP*) and type I collagen (*COL-I*)) will enable the acquisition of knowledge about the progression mechanisms of cell differentiation to better understand cell activities at different size scales during bone regeneration in in vitro scaffolds. These studies represent an essential step before proceeding to the formulation of preclinical studies for the development of translational research aimed at the creation of medical devices for clinical use.

## 5. Conclusions

In this study, the combination of fabrication techniques was explored, such as ES and 3D printing, as a strategy to overcome each technique’s limitations to form micro/nanostructured scaffolds that better mimic the complexity of ECM-like structures in hierarchically organized tissues, such as bone.

Coaxial electrospun PCL/Gt and PLA/Gt nanofibers were fabricated and used as coatings of 3D-printed scaffolds to validate their use for BTE.

It has been demonstrated that surface modification through the use of Gt-based coaxial nanofibers with peculiar morphological and physicochemical properties allows for the significantly influencing of the in vitro response of hFOB cells at the scaffold interface, improving adhesion, proliferation and in vitro bioactive response as indicated by increased mineral phase deposition and ALP activity.

In the light of collected biological results, Gt-based CNFs can efficiently serve as coatings to improve the in vitro response at the interface with 3D-printed PLA scaffolds. Advanced in vitro studies should be performed to quantify biochemical signals involved in bone regeneration process, as a propaedeutic step for the definition of preclinical models and future in vivo studies.

## Figures and Tables

**Table 1:** ES process parameters are used to manufacture nanofiber coatings.

Parameter	Single-Nozzle ^1^	Coaxial (Core/Shell) ^2^
Flow rate (mL h^−1^)	0.5	0.2/0.5 (inner/outer)
Voltage (kV)	10	10
Distance (mm)	140	140

^1^ Refers to PCL, PLA, or Gt solutions, separately. ^2^ Refers to PCL/Gt or PLA/Gt solutions.

## Data Availability

The original contributions presented in this study are included in the article; further inquiries can be directed to the corresponding authors.

## Supplementary Materials

The following supporting information can be downloaded at: https://www.mdpi.com/article/10.3390/biomimetics11050356/s1, Figure S1: 3D-printed PLA scaffolds, uncoated and coated with electrospun nanofibers of Gt, PCL, PLA, and coaxial nanofibers of PCL/Gt and PLA/Gt (red asterisk indicates the 3D-printed line zone). Scanning Electron Microscopy (SEM), SEI, 5000×. Figure S2. Voltage effect on Core-Shell structure formation for nanofibers of PCL/Gt and PLA/Gt synthesized at 8, 10, and 12 kV by coaxial electrospinning technique. High-resolution transmission electron microscopy (HR-TEM), SEI, 100,000×. Table S1: Average fiber diameter (AFD) and pore area for electrospun single-nozzle and coaxial-electrospun nanofibers. Table S2. Maximum degradation temperature (Tmax) for electrospun nanofiber coatings.
