# Supplementary material for: Evaluation of the Biological Response to Coating 3D-Printed PLA Scaffolds with Coaxial Gelatin-Based Electrospun Fibers
Source: Biomimetics (Basel). 2026 May 20;11(5):356. doi: 10.3390/biomimetics11050356 (PMC13204902; doi:10.3390/biomimetics11050356)
Supplement: Supplementary file 1 [file biomimetics-11-00356-s001.zip › biomimetics-4279279-supplementary.pdf]

SUPPLEMENTAL MATERIAL

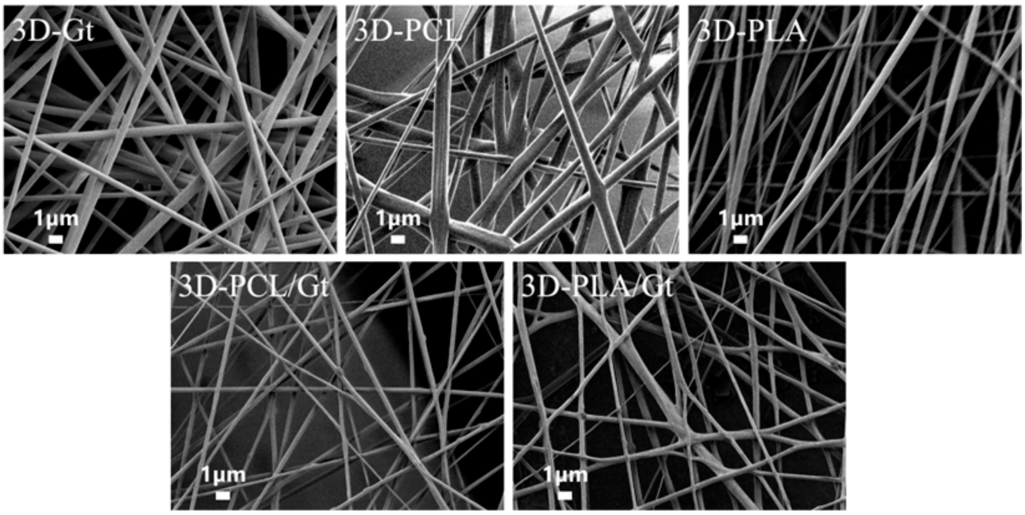

Supplementary-Figure S1. 3D-printed PLA scaffolds, uncoated and coated with electrospun nanofibers of Gt, PCL, PLA, and coaxial nanofibers of PCL/Gt and PLA/Gt (red asterisk indicates the 3D-printed line zone). Scanning Electron Microscopy (SEM), SEI, 5000x.

Supplementary Table S1. Average fiber diameter (AFD) and pore area for electrospun single-nozzle and coaxial-electrospun nanofibers.

**Nanofiber coatings**	**AFD (nm)**	**Pore area (μm^2)**	**Pore area (%)**
Gt	496 ± 128	0.88 ± 0.737	24.24 ± 6.71
PCL	375 ± 124	2.67 ± 1.83	22.10 ± 5.67
PLA	483 ± 199	2.39 ± 1.51	37.06 ± 6.43
PCL/Gt	278 ± 74	1.89 ± 0.81	48.54 ± 5.59
PLA/Gt	316 ± 124	1.41 ± 0.47	46.72 ± 5.97

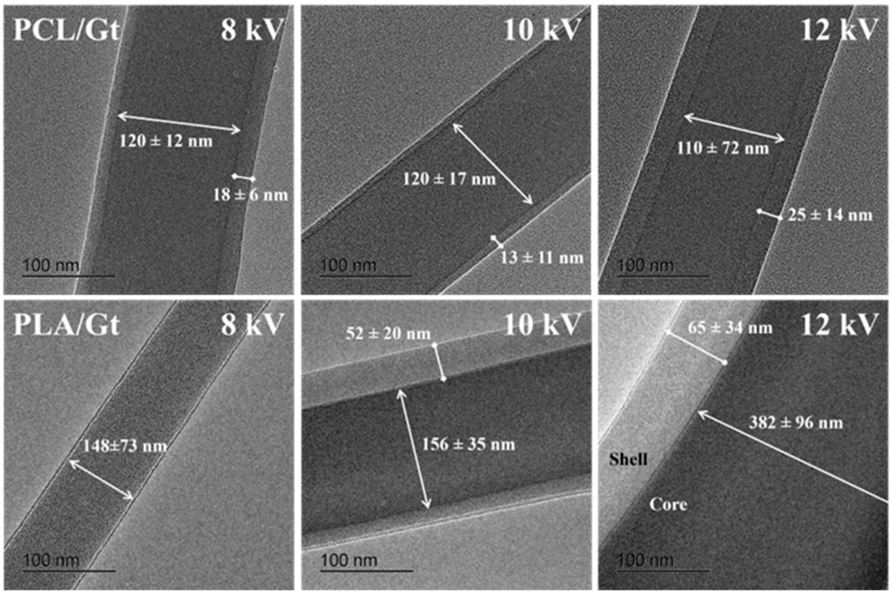

Supplementary-Figure S2. Voltage effect on Core-Shell structure formation for nanofibers of PCL/Gt and PLA/Gt synthesized at 8, 10, and 12 kV by coaxial electrospinning technique. High-resolution transmission electron microscopy (HR-TEM), SEI, 100,000x.

Supplementary - Table S2. Maximum degradation temperature (Tmax) for electrospun nanofiber coatings.

**Sample**	**Tmax (°C)**
PCL	405
PLA	318
Gt	313
PCL/Gt	320 and 397
PLA/Gt	319
